# Do Southern Hogs Have Trichinæ?

**Published:** 1882-01

**Authors:** 


DO SOUTHERN HOGS HAVE TRICHINÆ?—An investigation which seems to show that southern hogs do not have trichinæ was made by Dr. Jansen T. Payne, last summer. His report was submitted to the American Public Health Association, at its last meeting. In six months Dr. Payne examined 5,400 hogs, finding only 22 infected with the parasite in question.

The infected animals were reported as having been received from the following places: St. Louis, 18, Louisville, 2, and from the West, marked "unknown," 2, making a total of infected hogs, 22.

Of the hogs examined, only 529 came from St. Louis; most of them came from Louisiana (2,473), and Tennessee (1,060).

The observations lead to the belief, therefore, that southern-bred hogs are free from trichinæ. Still, such a deduction is not absolutely safe. If the fact were really proved, it would be one of great advantage to southern pork-raisers. Even as it is, they can profit from the fact that Tennessee and Louisiana hogs are almost entirely free from disease.

Incidentally, some other facts regarding the origin and communicatibility of trichinosis were developed. Observations seemed to show that hogs infect each other when enclosed in the same pen, and do not depend upon the rat as an intermediate host. The parasite is passed out of an infected animal along with undigested food, and the food is then eaten by a sound hog, who in turn becomes infected.

By Dr. Payne's examinations it was also ascertained that all the hogs infected with trichinæ were corn fed animals. No mast-fed animal was found to be infected.

The following sanitary regulations were recommended:

An improved trough should be made to contain the food of the hogs, and corn should not be scattered over the floors of the pens, as is customary at present, for by this means the corn finds lodgement in the dung, and thereby becomes a vehicle of infection. The pens should be kept very clean. When one hog of a lot is found to be infected with trichinæ, the animals should be separated, and not more than one hog placed in a pen.

By a rigid system of inspections Dr. Payne thinks it possible to prevent the sale of fresh pork infected by the trichinæ spiralis.

